# What Is the Contribution of Ia-Afference for Regulating Motor Output Variability during Standing?

**DOI:** 10.3389/fnhum.2017.00087

**Published:** 2017-03-02

**Authors:** Niklas König, Matteo G. Ferraro, Heiner Baur, William R. Taylor, Navrag B. Singh

**Affiliations:** ^1^Laboratory for Movement Biomechanics, Department of Health Sciences and Technology, Institute for Biomechanics, ETH ZürichZurich, Switzerland; ^2^Movement Laboratory, Department of Business, Health and Social Work, Bern University of Applied SciencesBern, Switzerland

**Keywords:** hoffman-reflex, postural sway, sample entropy, DFA, Lyapunov exponent, motor output variability, adaptive resource-sharing framework

## Abstract

Motor variability is an inherent feature of all human movements, and describes the system‘s stability and rigidity during the performance of functional motor tasks such as balancing. In order to ensure successful task execution, the nervous system is thought to be able to flexibly select the appropriate level of variability. However, it remains unknown which neurophysiological pathways are utilized for the control of motor output variability. In responding to natural variability (in this example sway), it is plausible that the neuro-physiological response to muscular elongation contributes to restoring a balanced upright posture. In this study, the postural sway of 18 healthy subjects was observed while their visual and mechano-sensory system was perturbed. Simultaneously, the contribution of Ia-afferent information for controlling the motor task was assessed by means of H-reflex. There was no association between postural sway and Ia-afference in the eyes open condition, however up to 4% of the effects of eye closure on the magnitude of sway can be compensated by increased reliance on Ia-afference. Increasing the biomechanical demands by adding up to 40% bodyweight around the trunk induced a specific sway response, such that the magnitude of sway remained unchanged but its dynamic structure became more regular and stable (by up to 18%). Such regular sway patterns have been associated with enhanced cognitive involvement in controlling motor tasks. It therefore appears that the nervous system applies different control strategies in response to the perturbations: The loss of visual information is compensated by increased reliance on other receptors; while the specific regular sway pattern associated with additional weight-bearing was independent of Ia-afferent information, suggesting the fundamental involvement of supraspinal centers for the control of motor output variability.

## Introduction

During standing, the human sensory motor system (HSMS) gathers sensory inputs from proprioceptive, vestibular and visual receptors, and continually transforms this information into the appropriate motor output (Prieto et al., 1996; Taube et al., 2008a). However, the output neural signals and the resulting motor actions are never constant, but rather exhibit a certain level of variability (Singh et al., 2012a; König et al., 2016). While standing, variability is exhibited as the non-constant or fluctuating behavior of the body's center of mass (CoM) relative to the base of support (BoS), i.e., postural sway. Such motor variability might partly be explained by the noisy behavior at each and every stage of HSMS processes including e.g., sensory perception at the receptor, information transmission via neural signaling, or non-constant motor-neuron firing (Faisal et al., 2008). Interestingly, recent investigations indicate that motor variability is not detrimental for the quality of motor actions, but rather a prerequisite for successful motor performance. For example, during motor learning, a U-shaped relationship can be observed, with variability reducing through the early stages of learning a task toward low levels after sufficient practice, but final stabilizing on higher levels in expert performance (Harbourne and Stergiou, 2003; Wilson et al., 2008; Fetters, 2010; Kyvelidou et al., 2013). Furthermore, it has been shown that individuals are able to regulate the magnitude of variability based on the demands of the specific motor task at hand (Loram et al., 2001; Wu et al., 2014; Pekny et al., 2015). From a theoretical perspective it has also been shown that the optimal levels of motor variability are sufficiently low to ensure stability, but sufficiently large to avoid rigidity in motor performance (Stergiou et al., 2006). Together, these findings suggest that the HSMS exhibits adaptability in the regulation of motor output that is dependent upon task demands, rather than adopting e.g., a simple minimization-of-error-strategy, thus indicating that variability in motor outputs are at least partially under dynamic control of the HSMS.

In order to maintain balance, the HSMS requires central nervous system (voluntary) control capacity, as demonstrated by cognitive dual-task experiments influencing postural sway (Mitra and Fraizer, 2004; Taube et al., 2008a). Such experiments propose an adaptive resource-sharing framework claiming that flexible allocation of HSMS resources consider (a) the precision required for the task, (b) the quality of sensory information available, (c) the effort required to collect this information, and (d) whether there is the requirement for active attention (Mitra and Fraizer, 2004). Depending upon their relative importance, the HSMS can regulate motor variability in order to prioritize for example, stability (i.e., by reducing the level of postural sway) or flexibility (i.e., releasing tight control of postural sway to free resources for other tasks). However, it remains to be investigated whether the HSMS uses different motor control strategies in order to maintain suitable levels of variability based on the demands of a particular task.

Within the framework of a biomechanical inverted pendulum model, an efficient feedback control mechanism for maintaining balance, particularly when focus on other tasks is required, could be provided by monosynaptic Ia-afference (Peterka and Loughlin, 2004). This neuro-physiological mechanism causes innervation of a muscle after stretch to its muscle spindles (e.g., due to sway) (Zehr, 2002). However, this reflex-loop, including the efferent neuro-muscular response, is additionally regulated by presynaptic inhibition (PSI) through various supraspinal and spinal influences (Zehr, 2002; Knikou, 2008), thereby rendering this reflex-loop an adaptable control element instead of an invariant mechanism. For example, dynamic modulation of the reflex occurs during the gait cycle, where larger reflex responses fulfill the requirements for increased stability in the stance and reduced responses the necessity for mobility during the swing phase (Hodapp et al., 2007). Therefore, regulation of the Ia-afference-loop could be a potential mechanism for governing motor variability, as inhibition would lead to a relaxation in control (i.e., flexibility), while facilitation of this reflex would cause tighter control (i.e., stability) of postural sway, albeit maybe at a higher level of “resource” consumption (Todorov and Jordan, 2002). Following a perturbation to the HSMS (e.g., eyes closed), sway is known to increase (Singh et al., 2012b), but overall balance is still maintained. It is therefore plausible that the HSMS becomes more reliant on other forms of sensory input (e.g., vestibular and proprioception), but in order to maintain low control costs, a different level of optimum variability is established, which could be achieved through adaptive regulation of the Ia-afferent input. Measurement of the Hoffmann reflex (HR), under standardized conditions, is able to provide a quantification of synaptic transmission resulting from an electrical stimulus evoked at a peripheral nerve onto the α-Motor units (α-M). Here, change in the magnitude of the synaptic response indicates interference from other (e.g., cortical) nervous system entities. Such an approach would therefore allow an assessment of the contributions of Ia-afference on governing motor output variability (Pierrot-Deseilligny and Mazevet, 2000; Zehr, 2002; Knikou, 2008). Through observing postural sway and H-reflex during visual and mechano-sensory perturbations to the HSMS, the goal of this study was there-fore to establish the level of contribution of Ia-afference on the regulation of motor variability.

## Materials and methods

In this study, the regulation of variability was assessed by measuring postural sway and H-reflex from the soleus-muscle (SO) in healthy adults while performing tasks under visual and mechano-sensory perturbations. In the first task, postural sway was perturbed by occluding visual information. It was hypothesized—according to the re-weighting of sensory information principle (Peterka and Loughlin, 2004)—that the contribution of peripheral sensory information (as determined by the H-reflex) will increase when no visual information is available (i.e., during eye closure). In the second condition, additional load around the CoM was applied during the standing task. Since the muscular torque that is required to maintain stability in the inverted pendulum model is characterized by the relationship between the lever arm (i.e., distance of the vertical projection of the CoM relative to the BoS) and body-mass (Robinovitch et al., 2002; Winter et al., 2003), a reasonable control strategy to avoid increasing muscular demands under increased body-weight would be to avoid the occurrence of large lever arms, i.e., maintain the CoM closer to the center of the BoS. This can effectively be realized by lowering the magnitude of postural sway, which likely suggests the requirement for tighter control (or increased precision) of the postural sway (i.e., stability) strategy. In order to better understand this mechanism, we have therefore explored the contribution of Ia-afference for regulating postural sway during the loaded limb condition.

Twenty-five healthy volunteers were recruited from the local community. Inclusion criteria were healthy physical and mental states. In seven subjects the experiment could not be performed, because subjects were either unable to tolerate the necessary stimulation intensities or the electrode location could not be properly identified to clearly elicit an H-reflex response. As a result, 18 participants (9 females and 9 males, age 26 (±4) years, height 175 (±7) cm, and weight 71.7 (±9.1) kg) were analyzed. The study was approved by the institutional ethics committee and was performed in accordance with the Declaration of Helsinki, and all subjects provided written, informed consent prior to participation in the study.

Each participant performed six different standing conditions in a randomized order; normal standing with eyes open (EO), standing with eyes closed (EC), standing with additional 20 and 40% bodyweight with eyes open (EO-BW20 and EO-BW40) and with eyes closed conditions (EC-BW20 and EC-BW40). To achieve the appropriate loading, vests with attachable sandbags (Gorilla Sports, Switzerland) were placed symmetrically around the participant's upper body.

During each of these six different conditions, participants stood barefoot on a force plate (Kistler, Winterthur, Switzerland; sampling frequency 1000 Hz), with feet together and hands crossed in front of their chest. Each participant was requested to focus on a black circle (15 cm diameter) placed 5 m anteriorly at eye level, and was asked to stand as still as possible. After testing each condition, the participants were given a short break of up to 5 min to prevent fatigue. The total duration of the experimental session was ~60 min.

### HR measurements

Before placing the EMG or stimulation electrodes, relevant skin areas were shaved, abraded with preparation gel (Nuprep, NR Sign Inc., Canada), and cleaned with water in order to ensure a low skin impedance of <1 kΩ (Hermens, 1999). The wireless EMG electrodes (Trigno, Delsys, United States) were placed on the SO according to the SENIAM protocol (Hermens, 1999). The HR stimulation electrode was attached while the subjects assumed a prone position. The cathode (1 cm diameter, Hellige, GE medical systems, Germany) was moved within the popliteal fossa of the right leg, until the largest H-response without an M-response could be evoked (Palmieri et al., 2004). Once located, this area was marked and the electrode was fixed with tape and an elastic bandage to prevent relative movement during the measurement. The anode (Spes Medica, 40 × 90 mm, Italy) was placed 2 cm above the patella.

As HR response depends on body position during postural sway (Tokuno et al., 2008), the mean sway position in the anterior-posterior (AP) direction from the first minute of every condition, was set as the sway-threshold (Nexus, VICON, United Kingdom) in order to trigger the HR-stimulation. Subjects were stimulated using a constant-current stimulator (DS7A, Digitimer, United Kingdom), which was only triggered when the participant swayed in a forward direction crossing the sway-threshold value, thereby ensuring similar muscle geometry in each stimulation and condition. Additionally, a minimal inter-stimulus interval of 8 s was used to avoid post activation depression (Chen and Zhou, 2011). To obtain full a H/M-recruitment curve, stimulus intensity (0.5 ms square-wave-pulses) was increased in increments of 0.5 mA around the H-max and M-max until they could clearly be identified.

**Figure 1:**
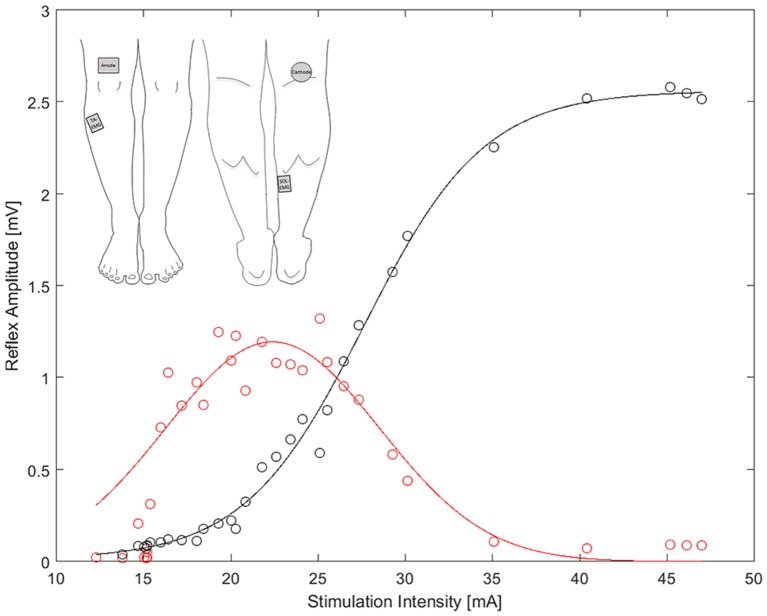
**Example of the sigmoid (black) and Gaussian (red) fit to evaluate H/M-recruitment curve**. Top-left: The position of the stimulation cathode and anode, as well as the EMG electrodes on the tibials and soleus muscle.

Sampling frequency of the EMG was set at 4 kHz and the signal was band-pass filtered (10–500 Hz, Butterworth 2nd order). The recording window to obtain the background EMG (bEMG), was set at 50 ms prior to each stimulus (Knikou, 2008). bEMG recordings were processed in the Imago software (IMAGO, pfitec, Germany) and extracted as root mean squares (RMS) values of the full rectified signal. The maximum H and M responses were assessed offline using a custom Matlab code (Matlab, Mathworks, United States) and a Gaussian function was fitted to the recorded H-amplitudes to receive a more robust H-response (Figure 1). Each function was weighted, with higher weights for the largest 30% of H-amplitudes, in order to best fit the curve over the region of interest (i.e., max H-responses) and thus most accurately determine the H-max value. Finally, a sigmoid function was used to fit the M-wave amplitudes for each condition (Brinkworth et al., 2007). The corresponding H-response at 20% of M-max was then identified for each condition and normalized to the bEMG (HR-bEMG). Thus, HR was represented as the gain of the reflex-loop, in order to address the problem of increasing SO-bEMG due to increasing weight and to allow standardized comparisons across the different experimental conditions (Palmieri et al., 2004).

### Postural sway measurements

**Figure 2:**
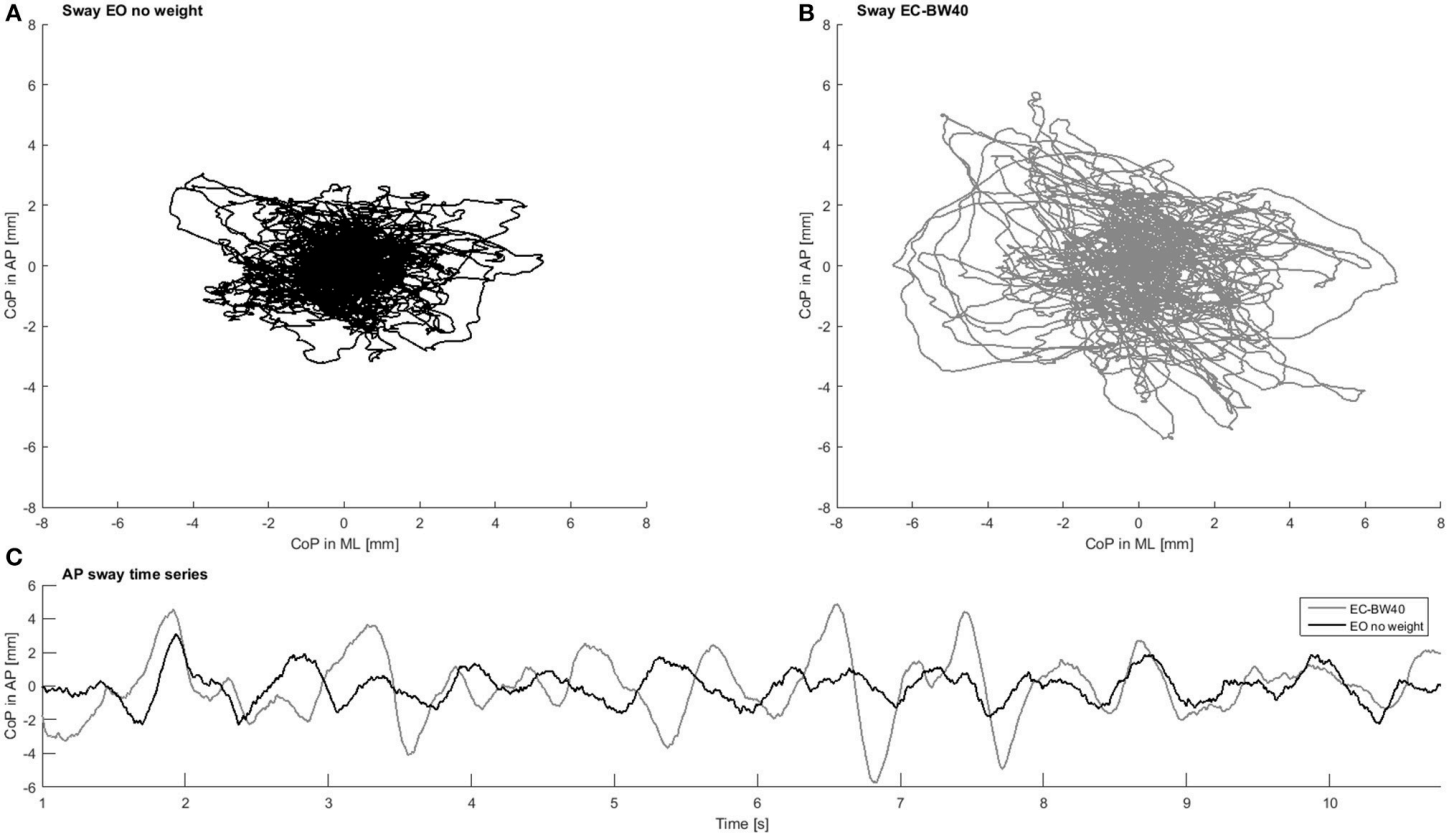
**Example CoP data in the presumably least challenging condition with eyes open and no additional weight (A)** and presumably most challenging condition with eyes closed and additional 40% body weight **(B)**. In **(C)** the time series of postural sway in anterior-posterior direction for the same two conditions is presented. Note the difference in sway magnitude and regularity between the two conditions.

Postural sway was measured during the initial minute of standing on the force plate, when no stimulation was applied. The first and last 7.5 s of each trial of the ground reaction force data were removed in order to avoid transients. Before calculating linear and frequency parameters, all data were band-pass filtered (0.75–35 Hz, Butterworth 4th order) and detrended. Parameters were calculated for the entire sway signal and the AP direction separately (Figure 2). Then, in order to quantify the magnitude of PS, multiple linear parameters were quantified, including sway area, velocity and distance of COP travel [a comprehensive list can be found in the literature (König et al., 2014)]. The frequency content of the signal was evaluated by assessing the absolute power within three frequency bands (low: <3 Hz; medium 3–10 Hz; high: 10–30 Hz). In addition, the temporal structure of sway was assessed using three non-linear parameters. Here, the raw-data (no filter applied) was down sampled (100 Hz), before the following parameters were calculated: Detrended fluctuation analysis (DFA) (Duarte and Sternad, 2008), sample (SE) and approximate entropy (AE) (Yentes et al., 2013) and largest Lyapunov exponent (LyE) (Ladislao and Fioretti, 2007). For the entropy measures, the input parameters (vector length m = 2; tolerance *r* = 0.2 x *SD*) were kept constant across all trials, after confirming that results were insensitive to other m/r-combinations. To determine LyE, the Wolf algorithm was used (Wolf et al., 1985), which requires the use of defined embedded dimensions (dim) and time lag (tau). Dim and tau were initially identified using the false nearest neighbor and average mutual information approaches for each trial separately. Lastly, constant values (dim = 5, tau = 5) were used based on their sample median (van Schooten et al., 2013) and applied for the final LyE calculation.

### Factor analysis

In total, 25 PS parameters were calculated. In order to reduce the dimensionality of the dataset, factor analysis (FA) using the “VARIMAX” procedure was applied, where each condition for all subjects (*n* = 18) was considered to be a case (108 cases). Kaiser-criterion was used to extract the appropriate number of components with Eigenvalues >1, while KMO criterion was used for determining sampling adequacy of the factor analysis. To ensure consistency of the original measure, the following criteria were applied in order to remove individual parameters from the analysis: (a) measures of sampling adequacy < 0.5, (b) measures with communality < 0.5, and (c) measures that caused complex structure (i.e., correlations >0.4 in two or more components). FA derived component z-scores were used for further analysis as well as for interpretation.

### Inferential statistics

Three mixed factor repeated measure ANCOVAs were conducted separately to explore the relationship between the dependent sway components on the independent measures of weight (three levels: No additional weight, 20 and 40% body weight) and vision (two levels: EO and EC) with normalized H-reflex at 20% M-max (HR-bEMG) as standardized Z-scores incorporated as the covariate. Inclusion of HR-bEMG as a covariate in the ANCOVA model allowed a direct contribution of H-reflex on the control of postural sway to be assessed. In addition, changes in motor control strategy could be identified between different standing conditions by investigating changes in the ANCOVA regression slopes. All statistical procedures were performed in SPSS (SPSS 23, IBM, United States) and alpha levels were set at 5%. *Post-hoc* comparisons were conducted using the Least Squares Differences (LSD) approach.

## Results

### Factor analysis

A total of seven iterations were required before obtaining the final three components, which were loaded with a total of 15 parameters (Table 1). These were interpreted as linear sway component (LSC), non-linear sway component (NSC), and frequency sway component (FSC). Since NSC was loaded with the positively correlated measures of entropy and LyE, they were interpreted as larger NSC values reflecting both irregular (based on entropy measures) and unstable (based on LyE) postural sway. These three components explained 92.6% of the total variance of the entire dataset (KMO = 0.846, Bartlett-Test of sphericity < 0.001).

**Table 1:** **Summary of the retrieved PCA components, displaying the communalities, explained variance by the components, and the loading of the different measures on the component**.

**Parameter**	**LSC**	**NSC**	**FSC**	**Communalities**
rel. SA	0.940			0.953
ellip. SA	0.977			0.976
rmsDist	0.955			0.943
rmsDist-AP	0.953			0.929
meanDist	0.961			0.956
meanDist-AP	0.955			0.932
lowFreq	0.971			0.965
mediumFreq	0.926			0.890
highFreq			0.938	0.889
lowFreq-AP	0.961			0.932
mediumFreq-AP	0.896			0.864
highFreq-AP			0.787	0.815
SE-AP		0.963		0.977
AE-AP		0.962		0.972
LyE-AP		0.945		0.897
Total variance explained (%)	64.436	20.156	8.004	

*Only loadings >0.4 are displayed. Abs, absolute; rel, relative; ellip, elliptical; SA, sway area; Dist, distance; Vel, Velocity; Freq, frequency; AP, Antero-posterior direction*.

### ANCOVA

**Figure 3:**
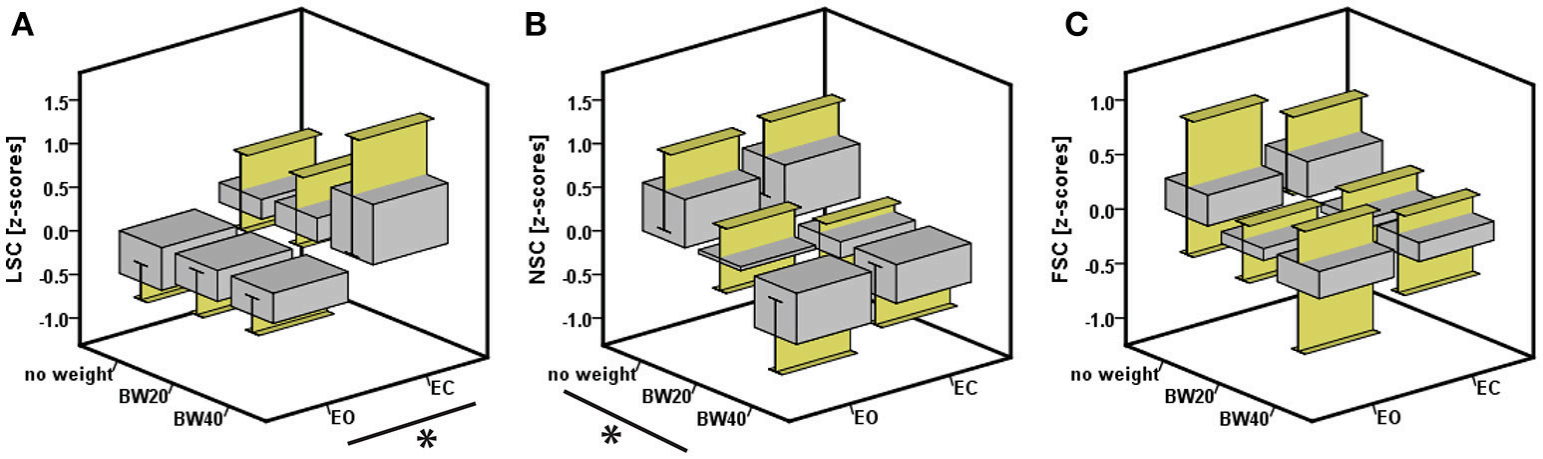
**Effect of vision and weight on the three dependent variables (A)** linear sway component (indicative of sway magnitude), **(B)** non-linear sway component (indicative of sway regularity), and (C) frequency sway component (indicative of sway periodicity). Asterisk indicates significant effects at *p* < 0.05.

**Figure 4:**
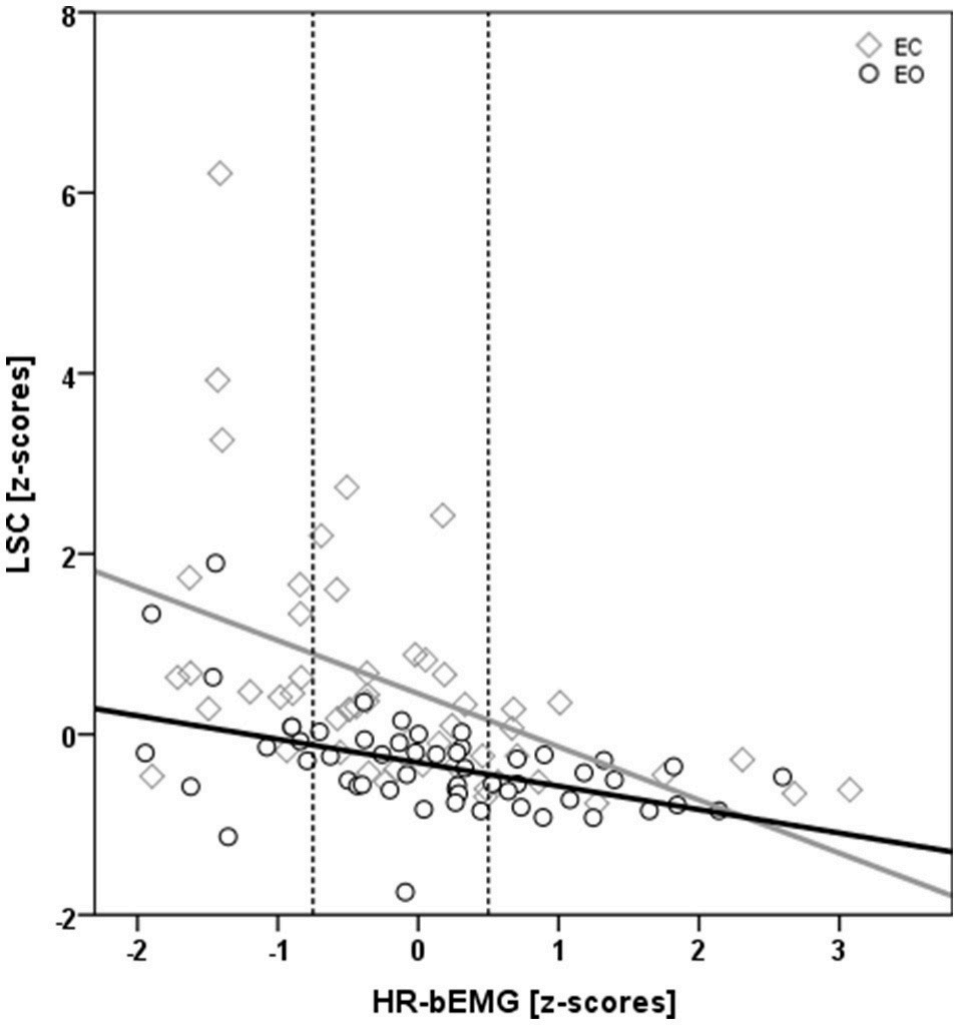
Scatter plot displaying the interactive effect of vision^*^ HR-bEMG on LSC. Both LSC and HR-bEMG have been converted to standardized Z-scores. There was a significantly larger decrease (*p* = 0.04) in LSC at higher HR-bEMG values with eyes closed (yellow diamonds) than with eyes open (gray circles) condition. The dotted gray lines represent the 25th and the 75th %-ile HR-bEMG values.

The occlusion of vision led to a significant increase in LSC of 0.85 standardized (Z-) score values, which corresponded to an effect size of 0.17 (Table 2; Figure 3). Furthermore, there was an interactive effect size of 0.04 of vision and HR-bEMG on LSC. *Post-hoc* comparisons of this interactive effect showed that with increased HR-bEMG, the effect of vision on LSC was reduced (LSC z-score of 1.04 at 25th percent-ile HR-bEMG; 0.87 at 50th percent -ile HR-bEMG; 0.69 at 75th percent-ile HR-bEMG) (Figure 4). No effect of weight was observed on the LSC.

**Table 2:** **Results of the ANCOVA for repeated measures with the sway components as depended variable and the HR-bEMG as independent variable and weight and vision as fixed factor with SS, Sum of Squares; N-df, Numerator degrees of freedom; D-df, Denominator degrees of freedom; and η^2^G, Generalized eta-squared**.

**SOURCE**		**LSC**					**NSC**					**FSC**				
	**N-Df**	**SS**	**D-Df**	***F***	**SIG**	**$\eta_{\text{G}}^{2}$**	**SS**	**D-Df**	***F***	**SIG**	**$\eta_{\text{G}}^{2}$**	**SS**	**D-Df**	***F***	**SIG**	**$\eta_{\text{G}}^{2}$**
Weight	2	2.61	29.9	2.1	0.61	0.03	**18.5**	**49.1**	**19.9**	<**0.01**	**0.18**	6.30	40.9	2.4	0.1	0.05
Vision	1	**20.38**	**39.1**	**48.0**	<**0.01**	**0.17**	0.03	75.8	0.0	0.84	0	1.75	47.7	1.1	0.30	0.02
HR-bEMG	1	0.19	64.4	1.4	0.24	0	0.09	83.0	1.1	0.3	0	4.81	82.4	3.7	0.06	0.04
Weight × Vision	2	0.72	29.5	1.3	0.29	0.01	1.8	49.5	2.6	0.09	0.02	0.81	41.5	0.2	0.84	0
Weight × HR-bEMG	2	0.21	27.2	2.0	0.15	0	0.39	45.5	0.4	0.7	0	0.84	23.9	0.7	0.48	0
Vision × HR-bEMG	1	**4.1**	**39.0**	**9.2**	<**0.01**	**0.04**	0.01	70.2	0.0	1.0	0	0.51	31.2	0.7	0.40	0
Subject		48.67					44.23					37.56				
Error		27.10					32.98					70.89				
C. Total		124.36					104.87					117.57				

*Bold indicates significant effects at p < 0.05*.

The addition of weight led to significant decreases in NSC of 1.04 Z-score values at an effect size of 0.18 (Table 2). Specifically, additional bodyweight led to a more regular and stable sway pattern. No interactive effect of weight or HR-bEMG was observed on NSC. There were no significant effects of any independent variables on the FSC.

## Discussion

The aim of this study was to assess whether peripheral Ia-afference is utilized in the control of motor variability during standing, and further to quantify the contribution of this input. Such information would illustrate the flexibility with which the human sensorimotor system (HSMS) employs the available resources and their characteristics for the regulation of motor output variability. During unchallenged standing (i.e., EO) there was no contribution of Ia-afferent feedback on the magnitude of sway. However, when challenging the available resources (i.e., under EC conditions) enhanced reliance on Ia-afference during standing could partly (up to 4%) counter-balance the effects of eye closure. The observation of a general down-regulation of H-reflex during eyes closed standing and the low contribution to explain LSC indicates that other resources of the HSMS are involved in the control of postural sway during sensory challenging postural tasks.

In order to establish the contribution of Ia-afference for controlling motor variability, FA was firstly conducted on postural sway parameters for a comprehensive assessment of the standing task. Linear sway components, LSC, were interpreted to quantify the magnitude of motor variability, whereas non-linear sway components, NSC, quantified the dynamic structure of the sway time series (Donker et al., 2007; Harbourne et al., 2009). The third component, FSC, comprised of absolute power in the high frequency bands and thus represents periodicity of the sway signal. The independent nature of these data suggest that the magnitude, dynamic structure, and frequency of sway are all unique characteristics of balance performance (Harbourne et al., 2009). Finally, the mixed factors ANCOVA revealed the level of contribution of peripheral Ia-afferences toward the control of LSC (magnitude of sway), in particular when input from visual receptors was challenged. There was no contribution of peripheral Ia-afference on NSC and FSC. As the contribution of Ia-afference on postural sway was low, despite larger effects of the task-demands on the linear and non-linear sway parameter, we conclude that other resources within the HSMS must be more involved in the control of motor variability.

When visual input is perturbed or removed, and thereby the available sensory information for the control of sway is reduced, postural sway becomes larger in magnitude ((Prieto et al., 1996; Taube et al., 2008b)), which was also apparent in the significant effect of vision on LSC in this study. Previously it was shown that eye closure leads in parallel to an inhibition of the H-reflex (Hoffman and Koceja, 1995; Earles et al., 2000), which is in accordance with the results of the present study. Furthermore, our results indicate that the effect of removing vision on the magnitude of sway was related to the contribution of Ia-afferences, such that greater contribution of peripheral sensory information (i.e., larger HR-bEMG) diminishes the effect of vision on sway magnitude. While this contribution was small, it results from only a single muscle and one extremity. Further investigation is therefore required to establish whether a more complex interplay of this Ia-afference mechanism occurs throughout the neuromuscular system, and in other scenarios. Since the maximum M response remained unchanged over the different test conditions, we concluded that a constant number of Ia-afferents were stimulated and therefore that any changes of HR-bEMG were not related to variations in the experimental setting of the test afferent volley, but rather to the physiological mechanisms that act to depress the H-response (Palmieri et al., 2004; Knikou, 2008). The contribution of HR-bEMG on LSC during eye closure could therefore indicate a re-weighting of inputs during conditions with reduced sensory information in accordance with the hypothesis that, with removal of vision, balance is regulated with larger inputs from (or reliance on) the proprioceptive (as well as mechano-receptive) sources (Peterka and Loughlin, 2004; Taube et al., 2008b). In this context, mechano-receptive cutaneous receptors in the plantar foot have been identified as potentially effective sway controlling entities (Priplata et al., 2002; Zhou et al., 2016).

The addition of external load led to a main effect on the NSC (the dynamic structure of the motor output variability), such that increased external load led to decreased NSC values, indicating more regular/stable sway patterns, i.e., less flexibility and more rigidity (Table 2). This effect was larger with an average change in regularity between weight conditions of 18%. The NSC comprised of the three parameters: Sample entropy, approximate entropy, and largest Lyapunov exponent. The general positive correlation between entropy and LyE suggest that sway regularity (i.e., low entropy values) is associated with larger convergence of nearby sway trajectories or stability (i.e., low Lyapunov exponent). Similar relationships have also been reported for healthy and pathological subjects where an increased regularity/stability pattern was associated with worse motor performance, and interpreted as a lack of flexibility to control balance (Huisinga et al., 2012; Rigoldi et al., 2013; Schniepp et al., 2013). In the current study, a similar pattern was observed in the sense that additional load led to a more regular/stable (i.e., less flexible) sway pattern. Importantly, there was no effect of additional weight on the linear sway parameters, which supports the notion that the HSMS reacts to increased weight around the CoM by increasing the precision of postural sway, as seen in the constant sway magnitude (unchanged LCS) and a more rigid control pattern (reduced NSC). Such behavior however, was not achieved with support from Ia-afference input, as observed in the absent interaction effect of weight and HR-bEMG on NSC.

Increasing load on the body is considered to be a perturbation to the mechano-sensory system, affecting various receptors such as Golgi tendon organs, cutaneous receptors or joint receptors, all of which have been shown to be involved in the control of movement (Gravano et al., 2011). In particular, it has been reported that increased weight load during standing leads to a decrease of sensory information transmission from plantar cutaneous receptors (Lhomond et al., 2016), which are considered to be a relevant input for maintaining sufficient background muscle activity during balance control (Meyer et al., 2004). Therefore, additional load not only changed the task-demands but also challenged the mechano-receptive inputs, thereby potentially rendering such input “unreliable.” This might also explain why the increase in task precision was not achieved by enhanced reliance on Ia-afference information. Interestingly, the regularity of sway patterns has also been attributed to the amount of attention exercised for the postural task (Donker et al., 2007; Rigoldi et al., 2013; Schniepp et al., 2013), and it could therefore be argued that the increased regularity in the sway patterns observed in our study result from the increased attention exercised to respond to the rather unusual task of carrying up to 40% body weight. This suggests a possible influence of supraspinal centers for the control of motor output variability during increased precision requirements (e.g., loaded tasks). However, the reliance on supraspinal centers for regulating sway needs to be further investigated (e.g., under dual-task paradigm). Unlike the LSC, the main effect of vision, as well as the interactive effect of vision and HR-bEMG, were found to be non-significant on the NSC, indicating that the HSMS is unlikely to rely upon visual information to control the dynamic structure of motor variability.

In conclusion, output variability during complex motor tasks is characterized by magnitude, temporal structure, and frequency components. It has been shown that the HSMS can select the required motor variability depending on the task-demands. When the available sensory information is challenged, the HSMS reacts by re-weighting the remaining inputs and increasing its reliance on Ia-afferences, thus diminishing the effects of perturbations to the visual system on postural sway. However, the contribution of Ia-afferences on the control of motor variability was small, indicating the necessity for other control entities to be involved. It also appears that when precise control of a task is required, and Ia-afference information is rendered unreliable, enhanced involvement of supraspinal centers is likely to be preferred over other peripheral mechanisms for the regulation of motor output variability.

## Author contributions

NK, MF, HB, WT, and NS: Contributed to the conception and design, the acquisition, analysis, and interpretation of the data, the drafting of the article, the critical revising of the intellectual content and the final approval of this study.

### Conflict of interest statement

The authors declare that the research was conducted in the absence of any commercial or financial relationships that could be construed as a potential conflict of interest.
